# Lehmann’s Physiological Chemistry

**Published:** 1855-12

**Authors:** 


### LEHMANN'S PHYSIOLOGICAL CHEMISTRY.

THE name of Lehmann is familiar to all readers of the modern works on physiology. His researches and opinions are referred to continually on all questions of organic chemistry as of the highest authority, and his work is indispensable to every one who would make himself acquainted with the present state of that interesting science. The publishers, Messrs. Blanchard & Lea, have brought the profession under new obligations to them by affording them an opportunity of securing, at a reasonable price, so valuable a repository of scientific facts.

